# Study on the Influence of Aging Temperature on the Microstructure and Properties of Ti-38644 Metastable β-Type Titanium Alloy

**DOI:** 10.3390/ma18163825

**Published:** 2025-08-15

**Authors:** Peiyue Li, Xinqi Zhang, Xingyu Liu, Zhiqiang Li, Zhihua Sun, Jian Hao, Jinping Pan, Zhi Li, Zhihua Wang

**Affiliations:** 1 Luoyang Ship Material Research Institute, Luoyang 471000, China; zxq_7258@163.com (X.Z.); 15502427922@163.com (X.L.); lizhiqiang@725.com.cn (Z.L.); 15075913476@163.com (J.H.); 15940514610@163.com (J.P.); 18098752101@163.com (Z.L.); 18304170154@163.com (Z.W.); 2 School of Materials Science and Engineering, Northeastern University, Shenyang 110819, China; 17832956970@163.com

**Keywords:** Ti-38644 titanium alloy, aging treatment, mechanical properties, microstructure

## Abstract

This article investigates the precipitation behavior of the phases in metastable β-type titanium alloys (Ti-38644) and their significant impact on the mechanical properties. By manipulating various solid solution aging parameters, the morphology, quantity, and distribution of the αs phase can be optimized. After one hour of solid solution treatment at 760 °C, the alloy is predominantly composed of the β phase, with a higher concentration of aluminum at the grain boundaries compared to the interior of the grains. Subsequently, after ten hours of aging treatment at 450 °C and 470 °C, the needle-shaped αs phase preferentially precipitated at the grain boundaries. As the aging temperature increased to 470 °C, the area percentage of the αs phase rose from 42.36% to 57.34%, while its yield strength (σs) increased from 967 MPa at 450 °C to 1211 MPa at 470 °C. This increase in σs results from the combined effects of dislocation strengthening ($\sigma_{\rho }$) and precipitation hardening. This article provides a comprehensive theoretical analysis of the various factors that influence σs, offering valuable theoretical support for the development of heat treatment processes for the Ti-38644 titanium alloy.

## 1. Introduction

With the continuous advancement in science and technology, the aerospace industry faces an increasingly urgent demand for high-strength, lightweight structural materials. Titanium alloys are particularly notable for their exceptional mechanical properties, especially metastable β titanium alloys, which not only possess low density and high specific strength but also exhibit excellent corrosion resistance and good forging performance. These alloys have gradually emerged as key materials to replace traditional high-strength steel and aluminum-based alloys [1,2,3,4,5,6]. Among them, the titanium alloy Ti-38644 has garnered significant attention due to its outstanding performance and has been widely utilized in various sectors of the aviation industry, both domestically and internationally. By optimizing heat treatment parameters, the strength of this alloy can be significantly enhanced, with its tensile strength (σb) at room temperature exceeding 1200 MPa, making it an ideal material for manufacturing high-performance fasteners and springs [7,8,9]. Consequently, there is a growing demand for Ti-38644 titanium alloy wire materials, accompanied by increasingly stringent quality requirements.

Through research, it has been determined that the size, distribution, and volume fraction of precipitates in the Ti-38644 titanium alloy during aging treatment significantly impact its properties [10]. Consequently, researchers have conducted a comprehensive study on the solid solution aging treatment process of the Ti-38644 alloy under various parameters. Gao et al. [11] investigated the effects of aging process parameters on the microstructure and properties of the Ti-38644 titanium alloy, finding that as the aging temperature increased, the αs phase coarsened significantly, resulting in a decrease in σb. Shang et al. [12] examined the effects of solid solution and aging treatments on the microstructure and mechanical properties of Ti-38644 titanium alloy rods. They observed that, with rising aging temperatures, needle-shaped αs phases gradually precipitated within the microstructure, and the distribution of these αs phases in the β phase became more uniform. Guo et al. [13] explored the effects of rolling and heat treatment on the microstructure and properties of the Ti-38644 titanium alloy. By optimally adjusting the parameters at each stage, they significantly enhanced the overall properties of the alloy. Li et al. [14] studied the impact of the aging treatment on solid solution cold-drawn Ti-38644 titanium alloy, discovering that the alloy exhibited excellent strength and plasticity when aged at temperatures between 550 °C and 600 °C. Rhodes and Paton [15] found, in their study on the relationship between the microstructure and mechanical properties of the Ti-38644 alloy, that β’ phase precipitates formed after short-term aging at 350 °C and 500 °C, which slightly increased σb. Wagner et al. [16] conducted heat treatment on Ti-38644 at various aging temperatures and found that the precipitation of the αs phase in the β matrix occurred at temperatures ranging from 400 °C to 600 °C. Above approximately 500 °C, the distribution of the αs phase tended to be uneven, with the presence of precipitation-free zones. Schmidt et al. [17] performed aging treatment on Ti-38644 and discovered that, after single-stage aging, the formation of precipitation zones and the presence of a second phase, αGB (α Grain Boundary), at the grain boundaries were closely related to the aging time.

Although researchers have extensively studied the solid solution aging heat treatment of the Ti-38644 titanium alloy, most of these investigations have focused on hot-rolled bars or forgings [18], while the research on titanium alloy wires remains relatively limited. Due to its unidirectional large deformation, titanium alloy wire exhibits a unique microstructure. Therefore, it is particularly important to explore the mechanisms underlying the solid solution aging heat treatment of Ti-38644 titanium alloy wire. This study aims to gain a deeper understanding of the microstructural characteristics of the αs phase in Ti-38644 titanium alloy wire and its impact on the alloy’s mechanical properties, with a specific emphasis on the role of the αs phase in enhancing the yield strength (σs). Firstly, the microstructure and mechanical properties of the alloy after solid solution aging treatment were investigated, revealing the effects of varying aging temperatures on the morphology of the αs phase and their impact on the strength and hardness (HV) of the alloy. Secondly, modified relevant formulae were employed to explore in depth how the precipitation of the αs phase enhances the σs of the Ti-38644 titanium alloy, providing theoretical support for the development of heat treatment processes for this alloy.

## 2. Materials and Methods

### 2.1. Materials

The Ti-38644 alloy used in this study is a wire with a diameter of 4.14 mm, and its deformation is about 10%. Titanium alloy raw materials are provided by Luoyang Ship Material Research Institute in Luoyang, China and the 4.14 mm wire rod is processed and prepared by us, entrusted by the Institute. Through experimental determination, the chemical composition of the alloy is as shown in Table 1. The phase transition temperature of the alloy was measured to be 736 °C using a calculation method, as detailed in the following formula:

$$T_{\beta }=882^{\circ }C+\sum The\;content\;of\;each\;element\times The\;influence\;of\;this\;element\;on\;the\;\alpha +\beta /\beta \;phase\;transition\;point$$

In the formula, 882 °C represents the phase transition point of pure titanium.

**Figure 1:**
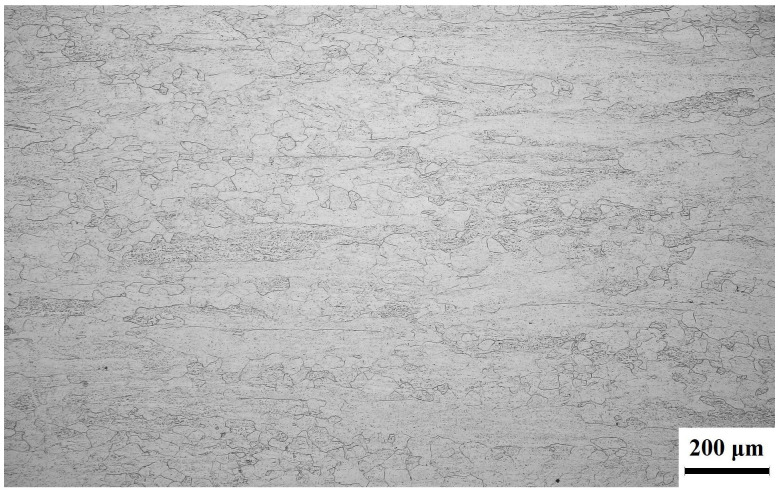
The microstructure of the alloy in its original state.

Figure 1 illustrates the microstructure of the Ti-38644 titanium alloy in its original state. The image clearly reveals that the microstructure is predominantly fibrous, with a small number of recrystallized grains.

### 2.2. Methods

This article discusses various solid solution aging heat treatments for alloys. First, the Ti-38644 alloy undergoes solid solution treatment at 760 °C for 1 h to ensure the complete integration of solute atoms into the matrix. Subsequently, aging treatment is performed at two temperatures, 450 °C and 470 °C, for 10 h each to promote the precipitation of nanoscale αs and enhance the strength of the titanium alloy. Additionally, the effects of aging temperature on the mechanical properties and microstructure of the Ti-38644 alloy are examined, with the heat treatment regime detailed in Table 2.

The AG-X100KN universal testing machine manufactured by Shimadzu Corporation in Kyoto, Japan was used to conduct tensile tests on the samples at a rate of 2 mm/min. To ensure the reliability of the results, each group of samples underwent three repeated trials. The HV-10Z micro Vickers hardness tester from Changzhou Sanfeng Instrument in Jiangsu Province, China, was employed to evaluate the hardness of the alloy. The hardness test was performed with a load of 5 kg and a loading duration of 10 s.

When preparing samples for optical microscope (OM) and scanning electron microscopy (SEM), the surface of the test specimen must be treated with both sandpaper and a polishing machine. The metallographic structure is examined using an OLYMPUS DSX500 optical microscope from Tokyo in Japan, and the sample undergoes scanning electron microscopy (SEM) inspection with a ZEISS GeminiSEM 460 scanning electron microscope manufactured by Carl Zeiss AG in Oberkochen, Germany. X-ray diffraction (XRD) is employed for phase analysis of the sample. The longitudinal section of the XRD sample is polished using 5000-grit sandpaper. The XRD equipment model is Smart Lab 9 kW, which is produced by Rigaku in Tokyo, Japan. The electron backscatter diffraction (EBSD) sample follows the same grinding and polishing process as the metallographic observation sample, but without etching. The equipment used is Crossbeam550 produced by Carl Zeiss AG. Transmission electron microscopy (TEM) analysis of the sample is performed using a JEM-F200 field emission transmission electron microscope from JEOL in Tokyo, Japan.

## 3. Results

### 3.1. Microstructure and Mechanical Properties of Alloys After Solid Solution Treatment

**Figure 2:**
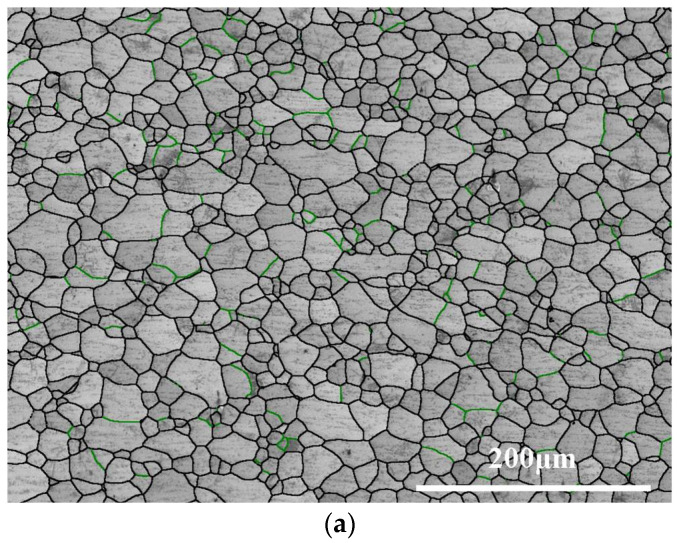

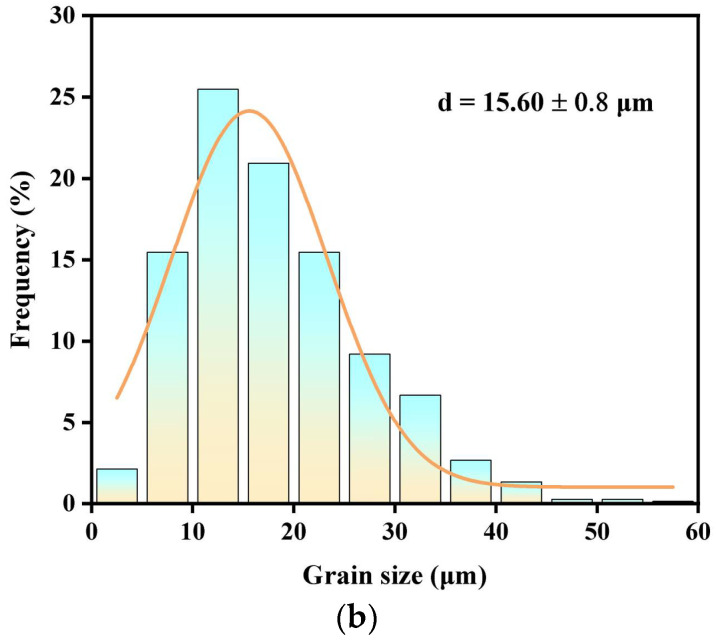
Microstructure of ST760 after solid solution treatment: (**a**) EBSD image of β equiaxed particles; and (**b**) β grain size distribution diagram.

The EBSD microstructure of the ST760 sample is illustrated in Figure 2. Following solid solution treatment, the ST760 underwent complete recrystallization and consisted of β equiaxed grains with an average grain size of 15.60 ± 0.8 μm (Figure 2a,b). As shown in the figures, no other identifiable precipitates were present in the β-phase matrix, indicating the absence of a second phase in the solid-solution-treated sample.

**Figure 3:**
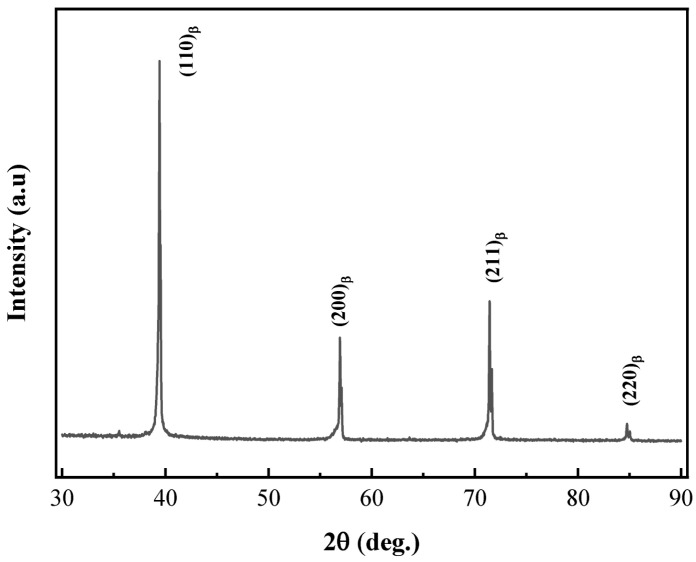
XRD spectrum of ST760 after solid solution treatment.

Figure 3 illustrates the XRD spectrum of the Ti-38644 alloy sample after undergoing solution heat treatment at 760 °C.

The spectrum shows that the primary peaks for the ST760 sample are (110) β, (200) β, (211) β, and (220) β, indicating a predominant presence of the β phase. Notably, no additional peaks are observed, further confirming the absence of any second-phase precipitates apart from the matrix following the solid solution treatment.

**Figure 4:**
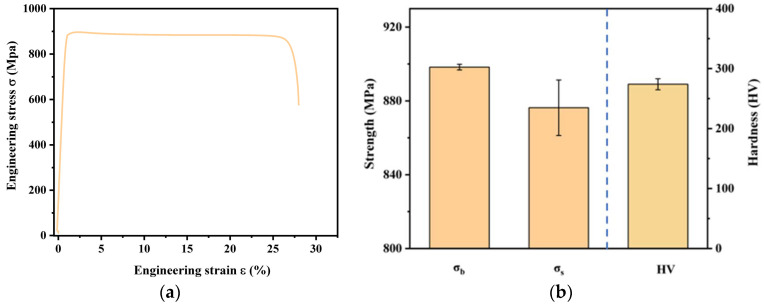
Mechanical properties of ST760 after solution treatment: (**a**) engineering stress–strain curve; and (**b**) strength and hardness.

Figure 4 illustrates the engineering stress–strain curve for the ST760 specimen. The specimen begins to yield at a strain of 0.2%. Following the yield point, the engineering stress experiences a slight decrease as the strain increases, and the stress decreases rapidly in the necking region prior to fracture. The strength and hardness properties of the ST760 specimen are presented in Figure 4b, with each result representing the average of three repeated tests. The σs with a 0.2% offset is 876 ± 15.0 MPa, the ultimate σb is 898 ± 1.5 MPa, and the Vickers hardness is 274.0 ± 9.1 HV 5.

### 3.2. Microstructure and Mechanical Properties of Alloy After Solid Solution Aging Treatment

**Figure 5:**
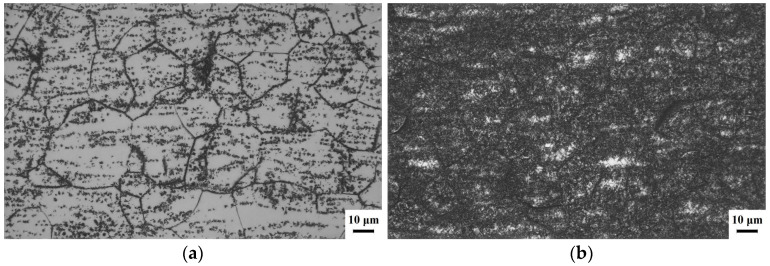
Light optical micrograph of Ti-38644 titanium alloy after aging treatment: (**a**) SA450; and (**b**) SA470.

The metallographic structures of the SA450 and SA470 samples are depicted in Figure 5. In Figure 5a, it is evident that the black second-phase αs precipitates are located at the grain boundaries and within the grains. The quantity of the αs-phase precipitation is relatively small and unevenly distributed. Figure 5b illustrates the metallographic structure at an aging temperature of 470 °C. It is clear from the figure that, compared to the sample aged at 450 °C, the amount of αs-phase precipitation significantly increases, accompanied by a rise in the volume fraction and a more uniform distribution. After rapid cooling in the single-phase region, the alloy attains a metastable β phase, which subsequently undergoes an αs transformation during low-temperature aging. The driving force for the phase transition from β to αs during aging arises from the difference in free energy between the metastable β phase and the αs phase at that temperature. At elevated temperatures, solute atoms possess higher thermal energy, which enhances the driving force for nucleation. Simultaneously, the diffusion rate of atoms significantly increases at 470 °C, allowing solute atoms to diffuse more rapidly from the matrix to the surface of the second-phase particles, thereby facilitating the precipitation of the second phase. Furthermore, the critical size for second-phase nucleation decreases at higher temperatures, increasing the likelihood of the nucleation process occurring. A comparison of Figure 5a,b reveals that the αs phase was not completely precipitated at 450 °C. However, as the temperature rises to 470 °C, the αs phase precipitates more thoroughly within the alloy matrix, and the precipitated αs phase gradually grows during the precipitation process.

**Figure 6:**
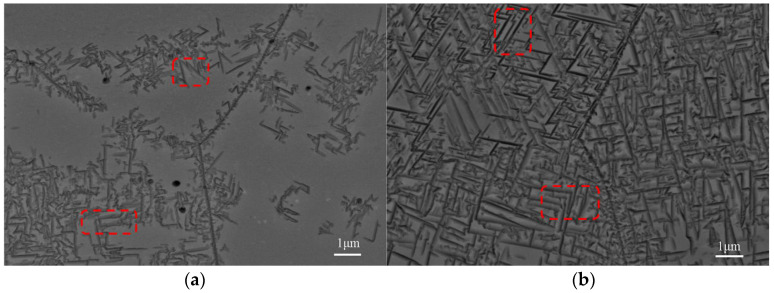
SEM microstructure of Ti-38644 titanium alloy after aging treatment: (**a**) SA450; and (**b**) SA470.

Figure 6 illustrates the SEM microstructure at aging temperatures of 450 °C and 470 °C. Small needle-like precipitates, identified as the αs phase, are visible. The secondary αs phases intersect both vertically and horizontally, exhibiting a parallel arrangement or forming specific angles, while adjacent αs phases typically display a triangular shape (as indicated in the red boxes). A comparison of the SA450 and SA470 samples reveals that the secondary phase preferentially accumulates along the grain boundaries.

**Figure 7:**
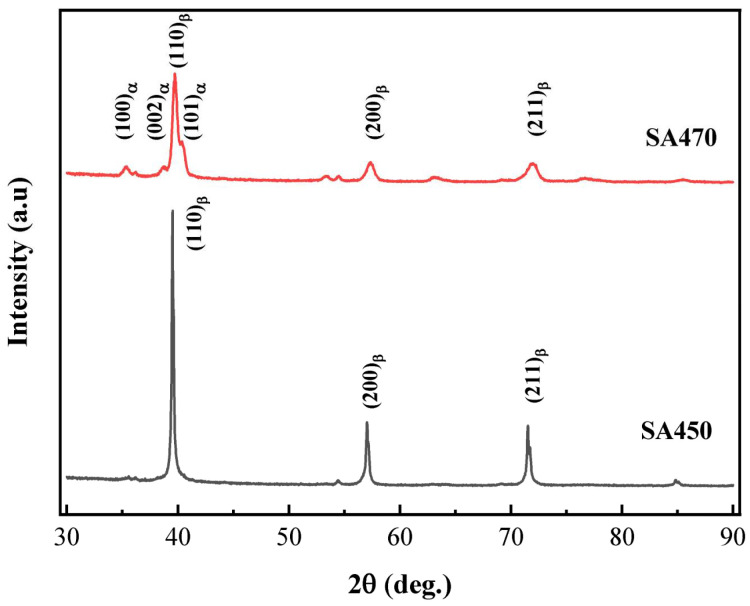
XRD spectra of the Ti-38644 titanium alloy after different aging treatments.

Figure 7 illustrates the XRD spectra of Ti-38644 alloy samples subjected to various aging heat treatments. The differing intensities and positions of the diffraction peaks across the spectra indicate a change in phase composition following heat treatment. The spectrum of the ST450 sample exhibits prominent peaks corresponding to the (110) β, (200) β, and (211) β planes, suggesting that it predominantly consists of the β phase. Metallographic and SEM images reveal that the ST450 sample also contains secondary αs precipitates; however, due to their low concentration, the corresponding peaks were not detected by XRD. In addition, the figure illustrates that the phases of the alloy after heat treatment at 470 °C primarily consist of the β phase, with a secondary precipitation of the αs phase. As the aging temperature increases, the diffraction peaks of the β phase and the αs precipitation phase in the spectrum of the ST470 sample exhibit significant changes. For instance, the (110) β diffraction peak decreases markedly in the ST470 °C sample, while the (100) α diffraction peak shows a substantial increase in intensity. This indicates that, during the aging process, the metastable β phase continues to decompose and undergoes a β → αs phase transition, resulting in a more complete precipitation and an increase in the volume fraction of secondary αs precipitates.

**Figure 8:**
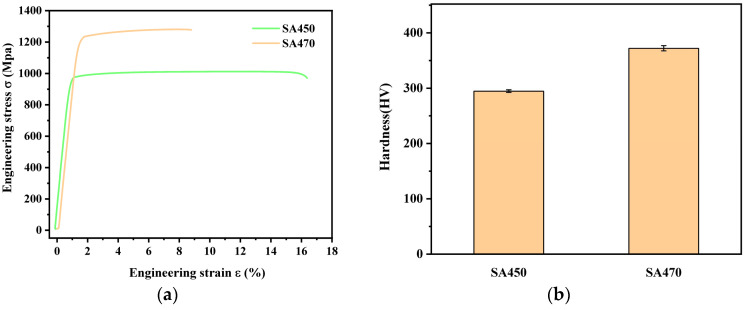
Mechanical properties of Ti-38644 titanium alloy after different aging treatments: (**a**) stress–strain curve; and (**b**) hardness.

Figure 8 illustrates the engineering stress–strain curves and Vickers hardness for the SA450 and SA470 samples. The figure reveals significant differences in the mechanical properties of the alloys subjected to varying aging temperatures. The σb and σs of the SA450 sample are 998 MPa and 967 MPa, respectively, while the SA470 sample exhibits σb and σs of 1297 MPa and 1211 MPa, respectively. Compared to the SA450 sample, the σb and σs of the SA470 sample increased by 29.96% and 25.23%, respectively. Furthermore, the stress–strain curve of the SA450 specimen demonstrates necking, indicating good plasticity, whereas the SA470 specimen fractures before necking occurs during the tensile test, suggesting poor plasticity. Consequently, a difference of 20 °C in the aging temperature results in significant variations in the strength and plasticity of the samples. In addition, after aging, the Vickers hardness of the alloy is 294.6 HV 5 at SA450 and 372.1 HV 5 at SA470, indicating significant differences in the alloy hardness after different aging processes. Table 3 reveals the mechanical properties of Ti-38644 after different heat treatments.

## 4. Discussion

### 4.1. Study on the Precipitation Behavior of αs Phase

During the heat treatment process of the Ti-38644 alloy, research groups led by Hu Ming [19] and Christ [20] discovered that aging results in the formation of an α-phase precipitation-free zone (PFZ), and the presence of this PFZ negatively impacts the mechanical properties of the alloy. During the aging treatment, Hu Ming et al. identified a non-αs-phase precipitation zone approximately 1 μm wide near the grain boundaries. This phenomenon occurs because, during the high-temperature quenching process, defects such as vacancies tend to diffuse toward the grain boundaries. This diffusion leads to a reduction in defects near the grain boundaries, adversely affecting the nucleation and growth of the αs phase in this region during subsequent aging processes. However, as illustrated in Figure 6, in this experiment, the αs phase preferentially precipitates at the grain boundaries. As the aging temperature increases, a significant amount of αs phase precipitates both within the crystal and at the grain boundaries. Notably, no precipitation-free zone of the α phase was observed during the aging process. To investigate the reasons for these discrepancies, further TEM testing was conducted on the ST760 sample.

**Figure 9:**
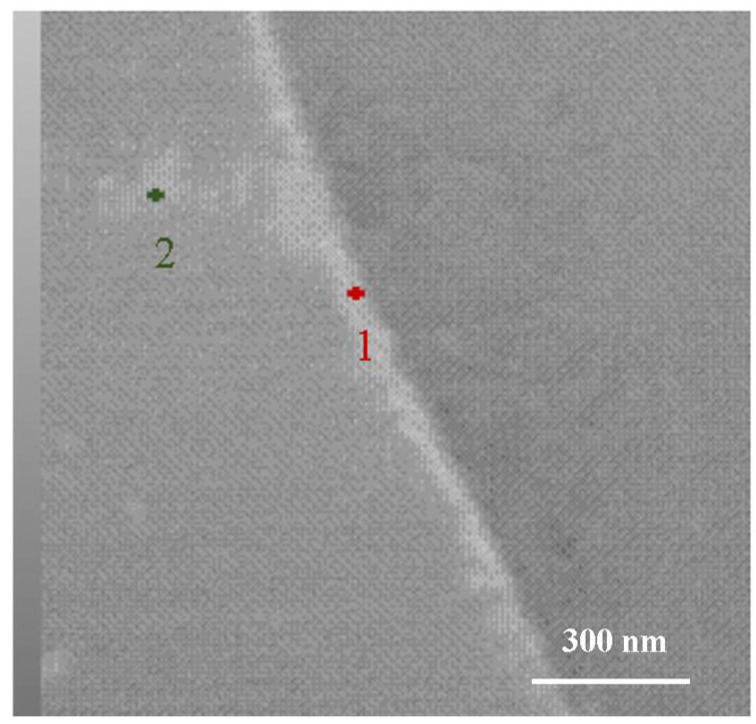
Element distribution point scan of ST760 sample.

Figure 9 and Table 4 present bright field images of ST760 samples at various magnifications, along with the scanning sites and proportions of elemental distribution points. Notably, there are distinct differences in composition between the grain boundaries (as shown in point 1) and the β matrix (as shown in point 2). The percentage of aluminum at the grain boundaries is slightly higher than that in the β matrix, while the percentages of molybdenum, chromium, and vanadium are slightly lower in the grain boundaries compared to the β matrix. Aluminum is a stable element in the αs phase, whereas molybdenum, chromium, and vanadium are stable in the β phase. This indicates that the stable elements in the αs phase tend to accumulate at the grain boundaries, while the stable elements in the β phase are more evenly distributed within the grain boundaries. This distribution provides a basis for the preferential precipitation of the αs phase at the grain boundaries.

In addition, the irregular arrangement of atoms at grain boundaries results in high interfacial energy, which leads to an imbalance in interatomic interactions. This high-energy state makes the grain boundary a favorable site for atomic diffusion and aggregation, causing the atoms at the grain boundary to be more active and diffuse more rapidly than those within the grain. Furthermore, the formation of a second phase requires the rearrangement and aggregation of atoms, and the high-energy environment at grain boundaries provides an additional driving force for this process. Consequently, under the combined influence of multiple factors, the αs phase preferentially precipitates at the grain boundaries.

Quantitative analysis was conducted on the area fraction of the αs phase, the width of the αs phase, and the spacing between adjacent αs phases in two sets of aging samples. This study employed multiple sets of SEM images for analysis. According to the statistical data of 10 pictures, variations in the aging temperature lead to significant changes in the proportion of the second-phase area. For the SA450 sample, the area percentage of the αs phase is approximately 42.36%. When the aging temperature increases to 470 °C, the area percentage of the αs phase rises significantly to about 57.34%. Concurrently, as the aging temperature increases, the size of the αs phase gradually expands. At an aging temperature of 450 °C, the width of the αs phase in the SA450 sample is approximately 88.56 nm, while, at 470 °C, the width increases to 107.03 nm. Due to the uneven distribution of the second phase in the SA450 sample after aging at 450 °C, areas of segregation and non-precipitation are observed. The spacing between the αs phases was analyzed using multiple sets of images, revealing a spacing of 8.24 μm. However, when the aging temperature reaches 470 °C, the precipitation of the second phase becomes more complete, the precipitate-free zone disappears, and the distribution of the αs phase becomes more uniform. An analysis indicates that the αs phase spacing in the SA470 sample is 369.13 nm.

### 4.2. The Effect of αs-Phase Precipitation on Enhancing Yield Strength

Guo et al. [18] have established a more systematic strength calculation model for titanium alloy bars, but have not yet extended the model to wire rods. Considering that there are significant differences between the wire rod and bar in the size effect, surface defects, and so on, if the original formula is directly used, it will produce large errors. Therefore, based on the work, the original formula is modified to accurately predict the strength performance of titanium alloy wire under different process conditions and significantly reduce the calculation error.

As illustrated in Figure 1 and Figure 8, the σs of the SA470 sample has significantly increased by 335 MPa compared to the ST760 sample. To clarify the specific role of the αs-phase precipitation on the σs of titanium alloys, we conducted a comprehensive theoretical analysis of the various factors influencing σs. According to the literature [21], the small second-phase αs plays a crucial role in the mechanical properties of the (α + β) two-phase titanium alloys. The hardness of the αs phase is considerably higher than that of the β matrix, causing the softer β matrix phase in the alloy to deform before the αs phase when subjected to stress.

Firstly, investigate the various factors that influence the σs of solid solution samples at 760 °C for 1 h. The σs of the ST760 specimen primarily results from lattice friction strength $\sigma_{0}$ (which includes solid solution strengthening), fine grain strengthening $\sigma_{H-P}$, and $\sigma_{\rho }$. Therefore, the theoretical yield strength $\sigma_{y}$ of the ST760 specimen can be calculated using Equation (1) [22,23]:

(1) $$\sigma_{y}=\sigma_{0}+\sigma_{H-P}+\sigma_{\rho }$$

The $\sigma_{0}$ of multicomponent alloys is influenced by the critical decomposition shear stress ($\sigma_{CRSS}$) of the pure titanium slip, along with displacement solution strengthening ($\sigma_{SSS}$) and interstitial solution strengthening ($\sigma_{ISS}$) in multicomponent titanium alloys [24,25,26]. Consequently, the $\sigma_{0}$ of these alloys can be expressed as follows [27,28,29]:

(2) $$\sigma_{0}=\sigma_{CRSS}+\sigma_{SSS}+\sigma_{ISS}$$

(3) $$\sigma_{SSS}={\left(\sum_{i}B_{i}^{3/2}X_{i}\right)}^{2/3}$$

(4) $$\sigma_{ISS}=0.02\mu_{\beta }(C_{o}{)}^{1/2}$$

Among them, the $\sigma_{CRSS}$ of pure titanium is approximately 180 MPa [28,30]. The variable $X_{i}$ represents the atomic percentage of solute elements in the alloy, while $B_{i}$ denotes the solute strengthening coefficient. This coefficient is influenced by the shear modulus mismatch and the lattice parameter mismatch between the substituent element and the solvent element [24,31] and can be referenced in the literature [28]. The shear modulus, denoted as $\mu_{\beta }$, has a value of 39 GPa, and $C_{o}$ indicates the atomic percentage of interstitial oxygen elements. Due to the interaction between the solute atoms and dislocations, solid solution strengthening serves as the primary strengthening mechanism in titanium alloys.

Grain boundary strengthening results from the interaction between the dislocation slip and grain boundaries, which hinder the movement of dislocations from one grain to another. As a result, refining the grain size can improve the strength of alloys. The relationship between grain boundary strengthening and the average grain size in ST760 can be described by the classical Hall–Petch (H-P) equation [31,32]:

(5) $$\sigma_{H-P}=k_{H-P}\cdot d^{-\frac{1}{2}}$$

Among these factors, d is the average grain size of the β matrix, which has been determined to be 15.6 μm, as illustrated in Figure 1. The theoretical H–P coefficient ($k_{H-P}$) for titanium alloys ranges from 0.5 to 0.7 MN·m^−3/2^ [23,33]. For the purposes of this article, a value of 0.6 MN·m^−3/2^ has been selected for $k_{H-P}$.

The $\sigma_{\rho }$ in the ST760 sample can be calculated using the Taylor model [34], which is expressed as follows:

(6) $$\sigma_{\rho }=\alpha M\mu_{\beta }b\sqrt{\rho }$$

In the equation, α is a constant that reflects the interaction between dislocations, and M is the Taylor factor, which has a value of 2.8 in β-titanium alloys. The symbol b represents the Burgers vector of the dislocation, with (b = 2.8 Å), and ρ denotes the dislocation density, which can be estimated from the peak intensity of XRD.

Based on a related formula, the XRD test results were analyzed, and the lattice parameters of the β phase were determined to be 0.332 nm, with a density (ρ) of approximately 3.79 × 1014 m^−2^. Consequently, the theoretical contributions of various factors to the σs of the ST760 specimen are as follows: $\sigma_{0}$= 530.3 MPa, $\sigma_{H-P}$ = 151.9 MPa, and $\sigma_{\rho }$ = 178.6 MPa. The $\sigma_{y}$ of ST760 is calculated to be 860.8 MPa, which closely aligns with the experimental value of 876 MPa.

Compared to the ST760 sample, the most significant difference in the microstructure of the sample aged at 760 °C for 1 h followed by 470 °C for 10 h is the precipitation of the small, uniform, high-density second-phase αs phase. These precipitated second phases not only enhance precipitation strengthening ($\sigma_{p-s}$) but also increase dislocation density due to the lattice distortion resulting from the β–αs phase transition. The high σs of the sample aged at 760 °C for 1 h and 470 °C for 10 h can be attributed to the presence of αs and the corresponding increase in dislocation density. Therefore, the increase in yield strength ($\Delta \sigma_{y}$) relative to ST760 at 760 °C for 1 h and 470 °C for 10 h can be estimated as the sum of the contributions from dislocation strengthening ($\Delta \sigma_{p}$) and αs $\Delta \sigma_{p}$ [21,35]:

(7) $$\Delta \sigma_{y}=\Delta \sigma_{p}+\sigma_{p-s}$$

Among them, $\Delta \sigma_{\rho }$ is caused by the difference in dislocation density between the β-matrix of the ST760 sample and that of the SA470 sample. $\Delta \sigma_{\rho }$ can be expressed as follows:

(8) $$\Delta \sigma_{\rho }=\sigma_{\rho }\left(\frac{300\;{}^{\circ}C}{100\;h}-520\;{}^{\circ}C\right)-\sigma_{\rho }\left(ST820\right)$$

By analyzing the XRD results at 760 °C for 1 h, followed by 470 °C for 10 h, the lattice parameters of the β and αs phases were determined to be a_β_ = 0.332 nm, a_α_ = 0.295 nm, and c_α_ = 0.479 nm, respectively. The dislocation density increased from 3.79 × 1014 m^−2^ in ST760 to 6.90 × 1014 m^−2^ at 760 °C for 1 h, followed by 470 °C for 10 h, confirming that the increase in dislocation density is related to the precipitation of αs. At this stage, the area percentages of the β phase and αs phase are 42.66% and 57.34%, respectively. By combining Equations (6) and (8), it is calculated that the $\sigma_{\rho }$ at 760 °C for 1 h, followed by 470 °C for 10 h, is 240.95 MPa, resulting in an increase of approximately 62.37 MPa in $\Delta \sigma_{\rho }$.

According to the research reports, uniformly dense and dispersed αs nano precipitates positively influence the strengthening of titanium alloys. To evaluate whether dislocations can transfer through the αs/β interface under the current stress conditions of the SA470 sample, we conducted theoretical calculations on the strength of the interface potential barrier ($\sigma_{IBS}$) of the αs/β matrix layer. The $\sigma_{IBS}$ consists of two components: the modulus mismatch ($\tau_{m}^{*}$) on both sides of the αs/β matrix interface and the lattice mismatch ($\tau_{\delta }^{*}$) of the slip system, resulting in an overall $\sigma_{IBS}$ [30,36]. The transfer of dislocations from one layer to another requires overcoming the $\sigma_{IBS}$ of these two components. Therefore, $\sigma_{IBS}$ can be expressed as follows [37]:

(9) $$\sigma_{IBS}=\tau_{m}^{*}+\tau_{\delta }^{*}$$

The discrepancy in the shear moduli between the αs and β phases hinders the movement of dislocations from the β matrix to the αs phase. The maximum stress resulting from this $\tau_{m}^{*}$ can be calculated using the Koehler equation [38]:

(10) $$\tau_{m}^{*}=\frac{\mu_{\alpha }-\mu_{\beta }}{\mu_{\alpha }+\mu_{\beta }}\frac{\mu_{\beta }\;\sin\theta }{8\pi }$$

Among them, $\mu_{\alpha }$ and $\mu_{\beta }$ are the shear moduli of the α and β phases, which are approximately 44 Gpa and 39 Gpa, respectively, in (α+β) titanium alloys [22,39]. The angle (θ) between the slip surface of the softer phase and the phase interface can vary due to the non-fixed orientation of the interface between adjacent phases, taking any value between 0 and 90 degrees. For our calculations, we use the median value of 45 degrees to determine the average angle. The interface between the αs and β matrices of titanium alloy exhibits a semi-coherent structure, where dislocations related to $\tau_{\delta }^{*}$ impede the transmission of dislocations. The stress required to overcome this barrier ($\tau_{\delta }^{*}$) can be calculated using the following equation [37,40]:

(11) $$\tau_{\delta }^{*}=\eta \mu_{a}(\frac{\delta_{a}}{a}-\frac{b}{W})$$

(12) $$\frac{\delta_{a}}{a}=\frac{a_{1}-a_{2}}{a_{2}}$$

where η is a constant valued at 0.5, and $\frac{\delta_{a}}{a}$ represents the $\tau_{\delta }^{*}$ constants of different slip systems on either side of the αs/β interface, which can be calculated using Equation (12). Among these, $a_{1}$ and $a_{2}$ are the lattice constants for the slip systems of the α and β phases, respectively, with values of $a_{1}$= 0.295 nm and $a_{2}$= 0.332 × (√3)/2 nm. The variable W denotes the average width of the αs phase. From this, it can be observed that the calculated σ_IBS at the αs/β interface are $\tau_{m}^{*}$ = ~66.1 MPa, $\tau_{\delta }^{*}$= ~512.39 MPa, and $\sigma_{IBS}$ = ~578.49 MPa. Therefore, it can be concluded that the primary contribution to the interfacial barrier strength arises from the $\tau_{\delta }^{*}$ of the slip systems on both sides of the αs/β interface.

Due to the hindrance of the dislocation transfer by the grain boundaries, dislocation pile-up can occur. Consequently, the coefficient k in the H–P relationship represents the extent to which grain boundaries impede dislocation movement. Eshelby et al. discovered that the accumulation of dislocations at the interface between two phases can also result in similar strengthening effects [41]. In this context, analogous to the H–P relationship, the interface reinforcement coefficient (k) is proposed as follows [37]:

(13) $$k=M(4\sigma_{IBS}\mu_{\beta }b/\omega \pi {)}^{1/2}$$

Among them, ω is a constant with a value of approximately 0.84. In this study, k was determined to be 172.99 MPa·(μm)^1/2^. Therefore, the increase in σs resulting from the $\sigma_{p-s}$ of αs can be calculated as follows [31,36]:

(14) $$\sigma_{p-s}=k\cdot \lambda^{-\frac{1}{2}}$$

Among the factors considered, λ is the average spacing between adjacent αs precipitates. According to the results presented in Figure 6b, the spacing of SA470 is determined to be approximately 369.13 nm. Consequently, the calculated value of $\sigma_{p-s}$ is approximately 284.73 MPa, which is significantly higher than that of $\Delta \sigma_{\rho }$. When considering both $\Delta \sigma_{\rho }$ and $\sigma_{p-s}$ factors simultaneously, the $\Delta \sigma_{y}$ increment caused by the precipitation of αs is 347.1 MPa. The $\sigma_{y}$ for the sample treated at 760 °C for 1 h, followed by 470 °C for 10 h, is 1207.8 MPa, which closely aligns with the experimental value of 1211 MPa, as shown in Figure 7.

A similar analysis was conducted on the sample at 760 °C for 1 h, followed by a temperature of 450 °C. The $\sigma_{y}$ of the sample at this temperature combination is approximately 974.8 MPa, with calculated values of 45.03 MPa and 68.95 MPa, respectively. This theoretical value closely aligns with the experimental data of 967 MPa.

**Figure 10:**
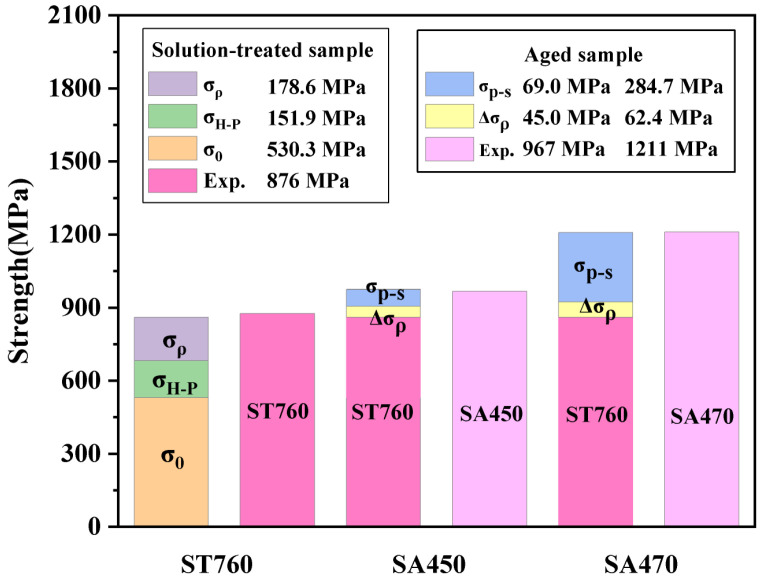
Individual contributions of different strengthening mechanisms to the yield strength of ST760, SA450, and SA470.

The individual contributions of each strengthening mechanism to the σs of ST760, SA450, and SA470 are illustrated in Figure 10.

## 5. Conclusions

1. After undergoing solid solution treatment at 760 °C, the Ti-38644 titanium alloy undergoes complete recrystallization, resulting in equiaxed grains of the β phase. An XRD analysis indicates that only the matrix β phase is present in the alloy at this stage, with a σb of 898 ± 1.5 MPa and a σs of 876 ± 15.0 MPa.
2. Through the analysis of the microstructure and mechanical properties of titanium alloys subjected to aging at various temperatures, it can be concluded that, at 450 °C, the αs phase appears as needle-shaped precipitates that are unevenly distributed, lacking distinct precipitation zones, and preferentially forms at grain boundaries. The area percentage of the αs phase is approximately 42.36%. When the aging temperature is increased to 470 °C, the amount of αs-phase precipitation increases and becomes uniformly distributed, with an area percentage of about 57.34% for the αs phase.
3. The substantial precipitation of the αs phase significantly enhances the mechanical properties of the alloy. Compared to the solid solution state alloy, the σs of the specimen treated at 760 °C for 1 h, followed by 470 °C for 10 h, increased by 335 MPa. For the specimens subjected to the treatment at 760 °C for 1 h, followed by 470 °C for 10 h, $\sigma_{p-s}$ (284.73 MPa) plays a major role in the increase in σs, in contrast to $\sigma_{\rho }$ (62.37 MPa).
4. The experimental results demonstrate that the alloy aged at 470 °C (1211 MPa) exhibits significantly improved mechanical properties compared to those processed at 450 °C (967 MPa). Consequently, for industrial applications, the aging treatment at 470 °C should be adoptedif a higher strength is desired.

## Figures and Tables

**Table 1:** Chemical composition of Ti-38644 titanium alloy (wt%).

Element	Ti	Al	V	Mo	Cr	Zr	Pb	Fe	O	N	C	H
Composition	Bal.	3.83	8.02	3.81	6.26	4.14	<0.010	0.097	0.08	0.007	0.009	0.0023

**Table 2:** Heat treatment parameter of Ti-38644 titanium alloy.

Heat Treatment	Heat Treatment Parameter ^1^
ST760	760 °C/1 h/WQ
SA450	760 °C/1 h/WQ + 450 °C/10 h/AC
SA470	760 °C/1 h/WQ + 470 °C/10 h/AC

^1^ WQ—water quenching, AC—air cooling.

**Table 3:** Mechanical properties of Ti-38644 after different heat treatments.

Sample Number	Tensile Strength σb (MPa)	Yield Strength σs (MPa)	Hardness (HV)
ST760	898 MPa	876 MPa	274.0 HV
SA450	998 MPa	967 MPa	294.6 HV
SA470	1297 MPa	1211 MPa	372.1 HV

**Table 4:** Scanning proportion of element distribution points in ST760 sample (wt%).

Element	Grain Boundaries/Atom%	Grain Interior/Atom%
Al	8.28	7.93
Mo	3.13	3.52
Cr	5.01	5.46
V	8.01	8.10

## Data Availability

The data presented in this study are openly available in the Materials Data Repository at https://mdr.nims.go.jp.
